# Alignment in the Hospital-Physician Relationship: A Qualitative Multiple Case Study of Medical Specialist Enterprises in the Netherlands

**DOI:** 10.34172/ijhpm.2022.6917

**Published:** 2023-01-22

**Authors:** Sander Ubels, Erik M. van Raaij

**Affiliations:** ^1^Radboud University Medical Centre, Nijmegen, The Netherlands.; ^2^Erasmus School of Health Policy & Management, Erasmus University, Rotterdam, The Netherlands.; ^3^Rotterdam School of Management, Erasmus University, Rotterdam, The Netherlands.

**Keywords:** Hospital-Physician Relationship, Interdisciplinary Collaboration, Contracts, Governance, Alignment, The Netherlands

## Abstract

**Background:** Policy-makers and hospital boards throughout the world have implemented different measures to create and sustain effective hospital-physician relationships. The ‘integrated funding’ policy reform in the Netherlands was aimed at increasing hospital-physician alignment and led to the unforeseen formation of medical specialist enterprises (MSEs): a fiscal entity representing all self-employed physicians in a hospital. It is unknown how hospitals and MSEs perceive their alignment and how they govern the relationship. This study explores the hospital-MSE relationship, and how governance styles influence perceived alignment in this relationship.

**Methods:** A multiple case study of five non-academic hospitals in the Netherlands was performed. Data was derived from two sources: (1) analysis of hospital-MSE contracts and (2) semi-structured interviews with hospital and MSE board members. Contracts were analysed using a predefined contract analysis template. Interview recordings were transcribed and subsequently coded using the sensitizing concepts approach.

**Results:** Contracts, relational characteristics, governance styles and perceived alignment differed substantially between cases. Two out of five contracts were prevention contracts, one was a mixed type, and two were promotion contracts. However, in all cases the contract played no role in the relationship. The use of incentives varied widely between the hospitals; most incentives were financial penalties. The governance style varied between contractual for two hospitals, mixed for one hospital and predominantly relational for two hospitals. Development of a shared business strategy was identified as an important driver of relational governance, which was perceived to boost alignment.

**Conclusion:** Large variation was observed regarding relational characteristics, governance and perceived alignment. MSE formation was perceived to have contributed to hospital-physician alignment by uniting physicians, boosting physicians’ managerial responsibilities, increasing financial alignment and developing shared business strategies. Relational governance was found to promote intensive collaboration between hospital and MSE, and thus may improve alignment in the hospital-physician relationship.

## Background

**Key Messages**

** Implications for policy makers**

- Integrated funding has contributed to hospital-physician alignment by (1) uniting physicians, (2) boosting physicians’ managerial responsibility, (3) increasing financial alignment between hospital and physicians, and (4) developing shared business strategies.
- Incentives may be used to prioritize certain projects/achievements. A combination of financial and non-financial incentives may be most effective.
- Relational governance may promote collaboration and development of a sustainable relationship; however, incongruent governance styles may increase risk of opportunistic behaviour.
- Our research suggests that creating a joint business strategy may be an important tool to develop relational governance and build a strategic partnership.

** Implications for the public**

The hospital-physician relationship has a major impact on quality of care and hospital performance. Whether or not physicians are employed by the hospital differs across countries and across hospitals. Boosting alignment of self-employed physicians with the hospital is a delicate process. Following healthcare reform in the Netherlands, Dutch self-employed physicians formed Medical Specialist Enterprises (MSEs). This study has explored the relationship between hospital and MSE and found substantial differences in the collaboration, governance and perceived alignment across Dutch hospitals. Higher alignment was perceived in hospitals where more relational governance (ie, informal, trusting, focus on mutual interest) was observed. Building a sustainable relationship by relational governance and creating shared strategy may result in a higher level of alignment and may possibly yield better hospital outcomes.

The relationship between a hospital and her doctors is of key importance for the provision of hospital care. The relationship influences the quality of care, cost-efficacy and financial results of a hospital.^1-3^ Following the *new public management* paradigm, hospital leadership has shifted from physician leadership to managerial leadership.^4,5^ Subsequently, in the face of growing healthcare expenditure and with the increasing managerial focus on quality, efficacy and accountability, an effective hospital-physician relationship has gained in importance to promote hospital performance.

###  Physician Employment

 There are largely two major forms in which physicians are employed: hospital-employed and self-employed. Historically, self-employed physicians travelled between different clinics to care for the ill.^5^ It was only when infirmaries grew larger, that physicians became more affiliated with one hospital. Globally there are large differences regarding the organisation of physicians and their employment: in the United Kingdom and Sweden all medical specialists are hospital-employed, in Germany, France, and Switzerland both employment-types are present but self-employed physicians are a minority, and in Canada and the United States the majority of physicians are self-employed.^6,7^ Self-employed physicians are present in countries with a centralized healthcare system and in countries with a decentralized healthcare system, in countries with a national healthcare fund and countries with social health insurance.^6^

 In the face of new public management, numerous policies and incentive programs have been initiated to enhance physician alignment and hospital performance.^6^ The traditional ‘doctor’s workshop’ has become obsolete and different forms of hospital-physician integration have been introduced.^8^ In addition, a shift towards hospital-employment has taken place, especially in the United States where the number of hospital-employed physicians has increased strongly since 2000.^9,10^ In a recent study, hospital-employment, an ultimate integration of the relationship between hospitals and their doctors, was associated with better hospital performance and cost reduction.^10^

###  Healthcare Reform in the Netherlands

 In line with the above-described international trend, the Dutch government commissioned a healthcare reform in 2015 aimed at increasing alignment between hospitals and self-employed physicians and enhancing hospital performance.^11^ In the Netherlands about half of medical specialists were self-employed at the time.^6,12^ All physicians working at academic hospitals (8 out of 69 hospitals in the country) have always been hospital-employed. In non-academic hospitals, 27% of physicians are hospital-employed.^12^ In most non-academic hospitals self-employed physicians work alongside hospital-employed physicians, the latter often from specialities such as paediatrics, geriatrics and rehabilitation medicine. Before 2015, the hospital and the self-employed medical specialists separately filed their claims with the healthcare insurer, leading to financial misalignment: if a surgeon could perform more procedures because of more expensive equipment, the surgeon would generate more income whereas the hospital would have to bear the cost for the equipment.

 In order to create more alignment between Dutch non-academic hospitals and self-employed physicians, the Dutch Healthcare Authority implemented a financial reform called ‘integrated funding’, ending the separate reimbursement for hospitals and medical specialists by January 2015.^13^ Only hospitals would file claims with insurers, and self-employed physicians were forced to make financial agreements with the hospitals related to their reimbursement and terms of payment.

 The reform was intended to enhance cost reduction, aid alignment of self-employed and hospital-employed physicians and eliminate barriers to selective contracting and value-base healthcare by healthcare insurers. Moreover, the Dutch government anticipated a shift towards hospital-employment and even offered a €100 000 subsidy for physicians who chose to become hospital-employed.^14^

 However, the reform had an unintended result: while only 5% of self-employed physicians chose to become hospital-employed,^15,16^ over 90% of self-employed physicians began establishing Medical Specialist Enterprises (MSEs).^13^ These enterprises were formed by uniting all the self-employed physicians of a hospital in one MSE, and each physician became an equal shareholder of the enterprise.

 The announcement of MSE formation led to a fierce debate. It was feared that the MSE would become a dominant body in the hospital, leading to subordinance of hospital-employed physicians.^17^ Also, the MSE was viewed as merely a fiscal construct to retain financial benefits.^18^ Proponents however underscored the opportunities for efficiency and innovation.^19,20^ In the years following the conception of MSEs, the debate remained active. While some proclaimed that the MSE had not become a dominant force,^21^ others protested that the MSE had paralysed hospital governance, that a lack of trust characterized the hospital-MSE relationship and that the hospital board had become dependent on the MSE and had lost its grip on the individual physician.^22-24^

 Whereas MSE formation was previously regarded as a temporary refuge in a transition towards a different physician-employment system,^25^ in 2016 the Dutch Healthcare Authority dissuaded any additional reform.^26^ As the MSEs have thus settled in the Dutch healthcare system, gaining insight into the hospital-MSE relationship is of great importance. Although some reports and publications have evaluated the integrated funding reform from an organisational and fiscal perspective,^13,26-28^ detailed insights into the new hospital-MSE relationship, its role in hospital governance and its contribution to alignment within the hospital are lacking.

 This study aims to investigate similarities and differences between five non-academic hospitals in the Netherlands in terms of hospital-MSE organisation, relationship, governance styles and alignment. The central research question of this study is: How do hospitals and MSEs perceive the hospital-MSE relationship and how do governance styles influence the perceived alignment in this relationship?

###  The Theoretical Background of the Hospit al-Medical Specialist Enterprise Relationship

 Alignment is a central concept in the hospital-physician and hospital-MSE relationship and is defined as “the degree to which physicians and organized delivery systems share the same mission and vision, goals and objectives, and strategies, and work toward their accomplishment.”^29^ In addition, alignment is not “imposing one’s will on the other,” but more a “mutual objective and the mutual willingness to work towards that objective.”^29,30^

 The way in which parties create alignment is called governance: “all actions that parties undertake to form and steer a relationship.”^31^ Two general governance styles can be distinguished: ‘hard’ contractual governance, in which contractual agreements and (financial) incentives are important to force another party in the desired direction, versus more ‘soft’ relational governance in which parties rely on the development of a relationship, and rely on trust and involvement to achieve their goals.^30,32-34^

 Both governance styles have a theoretical background in agency theory,^30,35^ which describes the relationship between a principal (hospital) and agent (MSE).^36^ As a principal delegates responsibility or decision-making power to the agent, a risk of opportunistic behaviour by the agent arises. Agency theory studies actions of the principal to direct the agent and limit the risk of opportunism.

 Contractual governance is in line with the traditional view of agency theory, which regards agents as a ‘homo economicus’ who attempts to maximize utility and may be steered by (financial) incentives.^30^ Principals relying on contractual governance will try to form a complete contract in which parties’ obligations, goals, contractual control and possible contingencies will be specified in detail.^30,37^ On the contrary, relational governance is in line with the more recent ‘social theory of agency.’^38^ This view relies less on (financial) opportunism, and describes the agency problem as a result of differing goals of an agent and principal. Subsequently, alignment may be created by intensive collaboration, building trust and promoting involvement.^39^

 The different theoretical views on the agency problem, and the associated styles of governance have been extensively studied in buyer-supplier relationships in a business-to-business context.^35,40^ Although the hospital-physician relationship has various similarities with the buyer-supplier relationship, the relationship is also inherently different. Consequently, some considerations may be noted when applying the agency problem and both theoretical views on the hospital-MSE relationship.

 In general, whereas parties in business-to-business situations may have the opportunity to switch to another buyer or supplier, in the hospital-physician relationship, switching is practically impossible.^8^ This is also true for the hospital-MSE relationship in the Dutch context, in which both parties are fully interdependent.

 Regarding the traditional agency lens, physicians are largely autonomous in their choice of patient treatment and hospitals have very little insight into what happens in the consultation room. Hospital reimbursement is predominantly based on prospectively priced diagnosis-related groups, and physician choices regarding treatment and product usage substantially influence revenues and operating margins of the hospital.^11^ Consequently, hospitals may perceive an increased risk of opportunistic behaviour by physicians due to asymmetry in knowledge and decision-making.^41^ Furthermore, physicians are the agents of two principals which may have conflicting interests: hospitals and patients.^41^ In the traditional agency view, the contract therefore should not only serve to limit opportunistic behaviour of a physician, but should also address possible conflicting interests stemming from the physician-patient relationship. Taken together, this may increase the use and importance of control mechanisms. Although the knowledge asymmetry complicates the control of the input or the process, control measures and incentives based on outcome might be effective elements in the buyer-supplier contract.^42^

 Moreover, some remarks should be made when applying the ‘social theory of agency’ lens on the hospital-MSE relationship. Dutch physicians tend to work at one hospital for a substantial part of their career, which may promote development of psychological ownership and a sense of involvement. However, some authors have argued that as physicians tend to have strong professional cohesion and internal control, physicians could feel more involved with their professional community rather than their hospital, which might impair the development of involvement and psychological ownership.^43,44^ Taken together, both views of agency theory may be applicable to the hospital-MSE relationship and may be used to better understand and recognize the mechanisms of governance in practice.

####  Governance Styles in Practice

 Contractual and relational governance have different practical characteristics, including contract types and communication styles, as summarized in Table 1. The contract is suggested to have a major impact on the subsequent relationship.^32,45,46^ A complete contract with detailed definitions of rights, duties, goals and incentives, has been described as a “prevention contract.”^30,37,45^ A contract which is purposefully left incomplete, leaving substantial room for relational governance, may be called a “promotion contract.”^45^ Such a contract is less detailed and goals will be described on a coordinating, collective level. The relational and emotional consequences of prevention and promotion contracts may differ widely.^45,47^ A prevention contract largely frames losses, while a promotion contract frames gains. Whereas the prevention contract formulates specific, minimal goals, ie, something that must be met, a promotion contract formulates more idealistic goals.^45^ Consequently, achieving a goal of a prevention contract leads to limited satisfaction and when a goal is missed it leads to high levels of disappointment.^48^ In addition, losing a financial incentive when not achieving a target can be perceived as a penalty. On the contrary, not meeting an idealistic goal of a promotion contract will exert little disappointment, but meeting a goal leads to great satisfaction.^48^

**Table 1:** Two Governance Styles for Hospital-Medical Specialist Enterprise Alignment

	**Contractual Governance**	**Relational Governance**
Contract	Prevention contract	Promotion contract
Perspective	Two distinct businesses	Joint business
Collaboration	Formal, at arm’s length	Informal, open
Communication	Binding	Making attractive
Control mechanism	Contract and incentives	Trust and involvement
Conflict resolution	Aimed at duties and rights	Aimed at common goals
Negotiation strategy	Distributive	Integrative

 Communication is also an important aspect of governance.^34^ In contractual governance communication is largely binding, where parties point out the others’ duties and rights.^34,46^ Parties have the perspective of two distinct companies. Consequently conflicts are experienced as a win-lose or zero sum,^46,49^ which leads to a rights-based approach and distributive negotiation, where both parties try to maximize their share.^50^ Also, parties are more inclined to rely on a third-party (eg, court, law firms) to resolve a conflict.^46,51^ The communication style that is more in line with relational governance underscores mutual interest and stimulates a collective perspective.^34^ Negotiations will be approached as a positive-sum game and parties are likely to adopt an interest-based and integrative negotiation strategy, where a collective perspective is used to look for a win-win solution.^46,50^

 The two distinct governance styles and related contract types and communication styles may be effective in different kinds of relationships. It has been argued that in relationships that require cooperative and flexible behaviour, relational governance with the use of promotion contracts leads to a more successful relationship.^45^ Moreover, while binding and controlling communication in line with contractual governance does reduce risk of opportunism, it is not likely to lead to a trusting relationship and may impede the development of an intensive, sustainable relationship.^34^ Relational governance underscores the joint potential of the relationship and promotes relational-specific investments and development of trust.^34,46^ Trust is an important requirement to develop a sustainable relationship.^52^ Trust may be defined as “the willingness of a party to be vulnerable to the actions of another party.”^32,53^ Distrust may be regarded as a “confident negative expectations regarding another’s conduct.”^32^ In current literature trust and distrust are regarded as two distinct constructs which can coexist.^32,54^ Both trust and distrust can have positive and negative outcomes^32^: trust stimulates transparency, openness, knowledge sharing and collective conflict resolution but may lead to overconfidence, over-embeddedness and a lack of objectivity; distrust stimulates vigilance and supports monitoring of vulnerabilities but may lead to rigidity, assuming harmful motives and developing fear.^55^ Thus, perceived trust and distrust may influence the governance style and likewise relational and contractual governance may influence trust and distrust.^32,34^ Whilst in contractual governance with a high level of contractual control some level of trust can be developed but outcomes are mixed, in relational governance using a promotion contract have been argued to increase trust and reduce distrust and opportunism, which may promote relational investments and relational value.^32,34^ Moreover, while both governance styles may create alignment, relational governance seems to be more effective when aiming to develop an sustainable relationship.^35^

**Figure 1:**
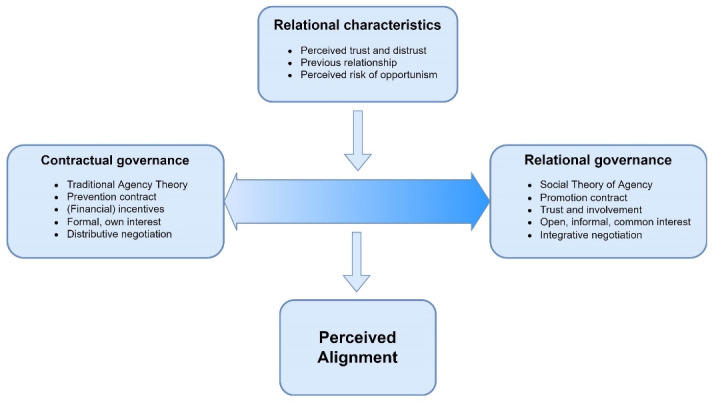
Conceptual Model of Governance and Perceived Alignment in the Hospital-Medical Specialist Enterprise Relationship.

 Taken together, one may expect that hospitals and MSEs which rely on relational governance will perceive higher levels of alignment. Our study is guided by a process model, displayed in Figure 1. Following the theoretical background, we expect that the perceived alignment between the hospital and MSE will be affected by the governance style which is used by both parties in a sustainable relationship. In turn, the governance style may be affected by relational characteristics such as the perceived risk of opportunism, perceptions of trust and other past experiences.

## Methods

###  Study Design

 The hospital-MSE relationship, governance styles and perceived alignment are investigated through a descriptive and exploratory study. Our object of study is the dyadic relationship between a hospital and an MSE. We aim to gain a ‘thick’ description of, and achieve detailed insight into, the hospital-MSE relationship and the attitudes and behaviours of the two contracting parties. The case study approach fits the objective to gain such rich insight.^56^ Instead of investigating a broad, diffuse sample, leading to a broad but shallow understanding, the case study applies more focus, using a smaller sample, leading to a more immersive, deep understanding of context, events, and opinions.^57^

 For this study, no approval by an ethical committee was required according to Dutch Law, as no personal data were collected, and participants were not subjected to any medical procedures. All participants consented with participation and participated voluntarily.

###  Case Selection

 As physicians in academic hospitals are all hospital-employed, this study focused on selecting non-academic hospitals were MSEs have formed. Given the sensitive topic, parties were approached through *convenience *and *snowball *sampling.^58,59^ In convenience sampling, cases are selected because they are accessible and available. Snowball sampling is a type of convenience sampling that relies on referrals from initial cases to generate additional cases.^59^ Hospitals from different regions and of different sizes were approached for participation to create a diverse case sample. Five hospitals and their MSEs consented to participate in this study. This sample size is sufficient to compare and perform a cross-case analysis, but not too large to be overwhelming.^57^

###  Data Collection

 Two types of data were obtained at the participating hospitals/MSEs: interview data and contract documents. Semi-structured interviews were conducted using a topic list as provided in Supplementary file 1.^58^ Per hospital two face-to-face interviews were conducted, one with a member of the hospital board and one with a member of the MSE board. All individual participants consented to participate in interviews and to have the entire interview audio recorded. All participating hospitals/MSEs consented to disclose their contracts. The contracts were obtained in paper or digital copy. Participants were offered to anonymize, blind of withhold certain paragraphs or appendices. Data gathering was conducted between February 2019 and May 2019. All data, both interview transcripts and contract documents were pseudonymized before data processing. All interviews and contract analyses were conducted by the same author (SU).

###  Research Variables

 This study focussed on the alignment between hospital and MSE as perceived by the hospital and MSE board members. Alignment is “the degree to which physicians and organized delivery systems share the same mission and vision, goals and objectives, and strategies, and work toward their accomplishment.”^29^ In different opinion articles in Dutch medical journals and magazines, the MSE formation was suggested to have a financial and strategic impact.^18-20,22-24^ In addition, the MSE formation was suggested to impact the relationships amongst physicians themselves (eg, self-employed and hospital-employed).^17,21^ Consequently, to investigate the overall alignment between hospitals and MSEs, three components of alignment were distinguished:

- *Strategic alignment*: Sharing a common mission, vision and strategy and working together to accomplish the mission.
- *Financial alignment*: The extent to which business models, costs, risks, and financial incentives are aligned and shared.
- *Alignment between physicians*: The extent to which the interests of all doctors, both hospital- and self-employed, are represented by the MSE, including the reimbursement model of individual physicians.

To assess the overall alignment within each hospital, first the three separate components of alignment were assessed based on contract analysis and interview data. Subsequently, the overall alignment was determined as an aggregate of the three components.

####  Interviews

 During the semi-structured interviews, the different types of alignment were investigated. Furthermore, views on the integrated funding and MSE formation, contract process, communication, use of incentives and perceptions of the relationship, trust, conflict resolution and hospital-specific context were covered during the interviews. A full overview of topics covered in the interviews can be found in Supplementary file 1.

####  Data Analysis

 Contracts were assessed on the extent to which alignment was described and established through contractual agreements. Contract components were categorized as prevention or promotion contracts using a contract analysis template (Supplementary file 2).

 Audio recordings of the interviews were transcribed using AmberScript transcription software. Transcripts were coded using the *sensitizing concepts *method.^60^ Predefined relevant concepts formed the basis of the coding process and included: alignment, trust, relationship, governance and context. All transcripts were first open coded separately, and overlapping codes were merged, followed by axial coding: an analytical process where relations, conditions, interactions and consequences were coded.^61^ In addition, codes were assigned to one of the sensitizing concepts and additional concepts were added (eg, constitution and transition) (Supplementary file 3).

 A *within-case *analysis was performed by examining and comparing findings of the separate analysis of the interviews and contracts within each case. Afterwards, a *cross-case* analysis was performed comparing the within-case analyses of the different hospitals, identifying similarities, differences and relationships. Coding and analysis were performed using ATLAS.ti 8.0 (Scientific Software Development GmbH, 2019, Berlin). This manuscript was written in line with the Standard Reporting in Qualitative Research and Consolidated criteria for reporting qualitative research guidelines.^62,63^

## Results

 After presenting characteristics of the five included cases, the findings will be presented in line with the process model: first, relational characteristics will be described, then governance styles and perceived alignment will be discussed.

###  Case Characteristics and Organization

 The size of the five included hospitals varied between 500 and 1000 inpatient beds. Different regions of the Netherlands were represented in the sample, two hospitals were in a predominantly urban environment with more hospitals nearby, while the other three hospitals were in a more rural area.

**Figure 2:**
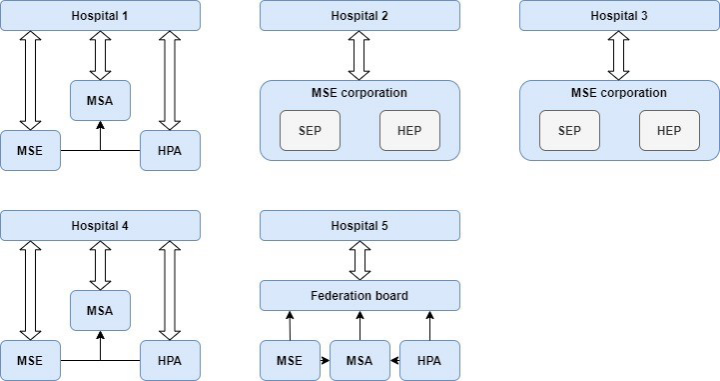
Hospital–Physician Relationships Per Case. Notes: The black arrows display the delegation of physicians to other physician parties. The bidirectional arrows represent lines of communication between the hospital board and physicians. Abbreviations: MSE, medical specialist enterprise; HPA, hospital-employed physician association; MSA, medical staff association; SEP, self-employed physicians; HEP, hospital-employed physicians.

 In line with hospital size, the size of the MSEs varied between 100 to almost 300 physicians. In two hospitals (case 2 and 3) the hospital-employed and self-employed physicians formed a joint corporation. In three hospitals (case 1, 4 and 5) the MSE only included self-employed physicians. In these cases, the hospital board had three different physician parties to communicate with (Figure 2): the MSE, the hospital-employed physicians association (HPA) and the medical staff association (formed by MSE and HPA representatives). Although on paper the three different parties had different topics to discuss, in practice the hospital boards experienced that they had to repetitively discuss many topics with all three parties. Regarding decision authority, MSEs were organised *bottom-up*, ie, the MSE board represented MSE members and were held accountable by MSE members, whereas the hospital boards could act *top-down* and were only held accountable by a supervisory board.

###  Relational Characteristics

 Regarding MSE formation, case 2 and 3 highlighted to have gone through an extensive preparation before the MSE was organized and the contract was drafted. Noteworthy, in these cases a joint MSE cooperation was formed, and the importance of the local context was emphasized: firstly, these hospitals were able to start preparations early because they were not hindered by disrupting events such as hospital mergers, regional competition, or intensified quality monitoring. Secondly, the hospital-physician relationship and relationship between physicians was perceived as stable before MSE formation, which enabled uniting all physicians in one cooperation.

 Following MSE formation, managerial responsibility had increased. Different MSE board members received managerial education and different MSEs had set up formal organisational structures including quality committees and human resources services. In addition, in case 1 uniting physicians in the MSE was remarked to have led to joint purchasing of medical goods (eg, surgical materials) by different specialties within the MSE.

 In all cases, the MSE was regarded as an important partner of the hospital board. The MSE office was often located close to the hospital board office. However, there were substantial differences in the level of trust. In case 2, 3 and 4, a high level of trust was perceived, but in case 1 and 5 lower trust and more distrust was perceived: “*We do have some informal contact [with the hospital board]. It is very dangerous when discussing alone with the hospital board as you must trust each other. [...] If you really want to arrange something, we must be more formal. We could coordinate some things with each other, but you should keep your cards close to your chest. There is no blind trust in each other, there is always some tension. I think this has to do with the history of the hospital and might be present in other hospitals too. I have always found it hard, naturally the hospital board has quite some power”* (MSE, case 5).

###  Governance

 The governance styles in the different cases were assessed based on the contracts, use of incentives and described collaboration.

####  Contracts

 The contracts of case 1, 2 and 3, and of case 4 and 5 showed many similarities. During the interviews different participants remarked that law firms offered draft hospital-MSE contracts. Presumably, the obtained contracts originate from two law firms.

 There was little difference between contracts regarding various topics such as termination, exclusivity, mediation & arbitrage and contractual control (Table 2). The main differences were the extent to which the contracts described the normative collaboration, shared business strategy and the introduction paragraph. The contracts of case 2, 3 and 4 described a shared mission, vision and business strategy, while in case 1 the “MSE obligated itself to the hospital’s business strategy” and in the contract of case 5 strategy was not mentioned. Similarly, whereas in the contracts of case 1 and 5 included no normative paragraphs, in case 2, 3 and 4 normative passages on the collaboration and relationship were found: togetherness, trust, equivalence and alignment. Lastly, especially in the contracts of case 2 and 3, an introduction described the importance of the hospital-MSE relationship, future directions, and the common goals and vision. Interestingly, interviewees mentioned that the hospital-MSE contracts played no important role once signed and were rarely consulted in any hospital, both in cases where contractual as well as relational governance was observed.

**Table 2:** Contract Analysis Per Case

**Items**	**Case 1**	**Case 2**	**Case 3**	**Case 4**	**Case 5**
Contract size (pages)	15	37	20	12	19
Appendices (number of pages)	6 (88)	6 (*)	4 (64)	*	>1 (*)
Contract duration	3 years	Undetermined	2 years	Undetermined	Undetermined
MSE reimbursement	Determined every year	% of hospital turnover	Not described	% of hospital turnover	% of hospital turnover
Internal MSE reimbursement	In line with hospital contract	“Stimulating alignment”	Controlled by hospital board	Not described	Not described
Performance indicators	Quality indicators	Not described	Not described	Quality and strategy	Quality
Use of incentives (type)	Some specified (penalties)	None specified	None specified	Many specified (mixed)	Some specified (penalty)
Conflict resolution	Mediation and arbitration	Mediation and arbitration	Mediation and arbitration	Mediation and arbitration	Arbitration
Strategy	MSE commits to hospital strategy	Shared mission and strategy	Shared strategy	Shared strategy	Not described
Normative paragraphs	Not described	Togetherness, collaboration, counsel, trust	Equivalence and alignment	Strive for extended future collaboration	Not described
Contractual perspective	Independent companies	Collectively towards high-quality care	Mixed collectively and independent	Predominantly collective	Independent companies
Contract type	Predominantly prevention contract	Promotion contract	Promotion contract	Mixed contract	Prevention contract

Abbreviation: MSE, medical specialist enterprise. * Unknown (size, number, or content).

####  Incentives

 Large variation was observed in the description and use of incentives. The hospital board of case 4 used incentives (ie, both financial and non-financial) widely to promote policy and quality development. An example of non-financial incentives is support of scientific projects by the hospital board. “*Everyone has his own stimulus which you have to look at. I am not saying that everything can be bought, however I think that [financial] incentives play an important role to further develop the organisation ” *(Hospital board, case 4).

 In case 1 and 5 financial penalties were formulated to stimulate specific quality developments such as complication registry and medical calamity investigations. MSEs regarded these incentives as a means to enforce specific developments. The hospital boards highlighted the mechanism of the incentives: “*[…] a transactional model. Regarding the financial incentives, we just pay them to do the calamity investigations [...] As long as we pay them by the hour, we can solve things. However, when we try to broaden the scope and explain it is also in their interest, then it becomes more difficult”* (Hospital board, case 1).

 Interestingly, in all hospitals that had put financial penalties on paper (ie, case 1, 4 and 5), the penalties were never put into practice. The hospital and MSE boards even knew that a penalty would never be executed, and the penalty incentive was only regarded as a means to put something on the agenda.

 In case 2 and 3 incentives were never or rarely used. The hospital boards remarked that the incentives did not fit their hospital culture and that they believed that (financial) incentives would not benefit a sustainable relationship.

####  Collaboration

 There were large differences regarding the collaboration between the hospital and MSE (Table 3). In some cases, the MSE predominantly guarded the interests of the physicians, while in other hospitals the MSE truly collaborated with the hospital to fulfil strategic goals and overcome future challenges: “*The coming years, the major challenge will be the government declaring zero growth of healthcare expenditure [...]. We must look for solutions together. […] I think this will be the major challenge the coming years. And we must rise to that challenge together, there is no other way”* (MSE, case 2).

**Table 3:** Hospital-Medical Specialist Enterprise Collaboration and Governance Style

**Item**	**Case 1**	**Case 2**	**Case 3**	**Case 4**	**Case 5**
Collaboration	Formal	Informal	Informal	Formal	Formal
Strategy	Independent	Shared	Shared	Shared	Independent
Perspective	Own interest	Shared interest	Shared interest	Shared interest	Own interest
Conflict resolution	Distributive	Integrative	Integrative	Mixed	Distributive
Governance style	Mostly contractual	Relational	Mostly relational	Mixed governance	Contractual

 In case 2, 3 and 4 a shared hospital-MSE business strategy played a crucial role. Drafting the strategy together led to openness between both parties and underscored their common goal. The shared strategy, and not the contract, was important to keep each other committed and the shared business strategy enabled the MSE to place the shared interest above the interest of the MSE or individual specialties: “*The hospital has a long-term strategy which is explicitly supported by physicians and the MSE, that is very important [...] without that strategy we would constantly bicker with each other, but the strategy requires us to choose for a quality policy, make uncomfortable choices and apply a certain remuneration system. If we did not have the shared strategy, we would not come to terms about these issues”* (MSE, case 4).

 In case 1 and 5 the hospital and MSE both had their own, separate business strategy. Whilst in hospitals with a shared strategy the collaboration was informal and close, in cases 1 and 5 the collaboration was more formal and at arm’s length. Both parties focused on their own interests and gains.

####  Governance Styles

 In case 1 and 5 predominantly contractual governance was observed. In these cases, the hospital and MSE remarked that the governance style was not always perceived as desirable or beneficial. Moreover, the hospital board of case 1 remarked that they attempted to resolve multiple conflicts using relational governance. However, despite the vulnerable stance and emphasis on the shared interest, the MSE – under pressure of the physicians – kept a contractual stance, focussing on their own short-term (financial) interests. The hospital board remarked that such ‘incongruence’ of governance styles could lead to increased opportunistic behaviour.

 The hospital board and MSE in case 2, 3 and 4 underscored that they were satisfied with the governance style in their relationship and remarked the governance style matched hospital and physician culture. The hospital board of case 4 chose to apply a mixed governance style, relying on incentives next to relational governance, because it fitted the entrepreneurial character of the MSE. While in case 1 and 5 the parties seemed to have no control over the governance style in the hospital-MSE relationship, in case 2, 3 and 4 the parties seemed to be able to influence the governance style.

###  Perceived Alignment

 In Table 4 the level of perceived alignment is presented. Strategic alignment was high or very high in case 2, 3 and 4, where a shared business strategy was observed. The extent to which the MSE positioned themselves as a strategic partner of the hospital board varied widely. In addition, hospital board members in case 1 and 4 felt that the role of the MSE board as representatives of the MSE slowed decision-making and in case 1 a lack of mandate of the MSE board was experienced.

**Table 4:** Hospital-Medical Specialist Enterprise Alignment

**Alignment**	**Case 1**	**Case 2**	**Case 3**	**Case 4**	**Case 5**
Overall	Moderate	Very high	High	High	Low
Strategic	Moderate	Very high	High	Very high	Low
Financial	Low	Moderate	Moderate	Moderate	Low
Between physicians	Moderate	High	High	Moderate	Moderate

 Financial alignment was low to moderate in all cases. Integrated funding had largely aligned hospital and MSE reimbursement. In different cases the alignment of hospital and MSE reimbursement was remarked to have contributed to joint investments, such as a hybrid operating room. However, in case 5 the integrated funding was experienced as a financial dependency rather than alignment: “*The MSE is dependent on the financial wellbeing of the hospital [...] It is like a shop within a mall. A shop can only benefit if people come to the mall [...] In that way we are tied to each other, through regulations, through the integrated funding”* (Hospital board, case 5).

 Although the MSE reimbursement scheme was contractually aligned with the business model of the hospital in cases 2, 3 and 4, there was a lack of alignment between the MSE reimbursement and the internal reimbursement scheme within the MSE in all five cases. All internal reimbursement schemes were based on an activity-based model, incentivizing production.

 Regarding alignment between physicians, in case 1, 4 and 5 the MSE experienced a moderate level of alignment amongst their physicians. However, the consequences for the MSE-hospital relationship varied. In case 1 and 5, the lack of alignment between physicians led to the MSE choosing for their own physicians’ interest in some instances, disrupting hospital-MSE collaboration, and in case 1, lack of authority and decision-making power of the MSE board was perceived as a major challenge. Although in case 4, low alignment between physicians was also perceived, the shared hospital-MSE strategy was remarked to prevent impact on the hospital-MSE relationship.

 Taken together, the perceived overall alignment was (very) high in case 2, 3 and 4, whilst in case 1 and 5 the perceived overall alignment was lower. Interestingly, the cases with higher alignment exhibited relatively more relational governance.

## Discussion

 This multiple case study has explored the relationship and perceived alignment between hospitals and MSEs. The hospital-MSE relationship and associated alignment was perceived very differently across the five cases, and different governance styles were observed. In hospitals where more relational governance was observed, a higher level of alignment was perceived.

 Relational characteristics and local context may have played an important role in shaping governance styles. In cases that perceived high alignment and relied on relational governance, the previous relationship was perceived as positive and there was more trust. In addition, these cases were remarked to not have been affected by disrupting events such as mergers.

 The integrated funding reform was intended to increase alignment between hospitals and physicians. The MSE formation was an unforeseen outcome when the reform took effect and the consequences for hospital-physician alignment were unclear. Despite the differences between the cases and exploratory study design, four mechanisms were highlighted in our study through which the formation of MSEs and the hospital-MSE relationships that ensued may have contributed to alignment: (1) uniting physicians, (2) boosting managerial responsibility, (3) increasing financial alignment between hospital and physicians, and (4) developing a shared business strategy. First, the MSE has united self-employed physicians and, in some hospitals, also included hospital-employed physicians in a joint cooperation. Uniting physicians has promoted their shared identity and development of common goals, similar to findings of previous research.^64,65^ For example, the alignment between physicians led to joint purchasing of medical goods (eg, surgical equipment) by different specialties.

 Second, MSE has increased the managerial responsibility of physicians. The MSE board has a more formal managerial position than physicians had before, and physician management further professionalized by educating MSE board members and setting up human resources services and physician-led quality committees. Taking up managerial tasks within the MSE was regarded as prestigious, which may have persuaded the physicians by promoting personal status and legitimacy.^66,67^ Creating more mandate may further promote alignment, as the lack of authority and decision-making power was regarded as an important bottleneck for further development of the hospital-MSE relationship.

 Third, a certain level of financial alignment was observed in all hospitals: the integrated funding reform itself aligned the MSE reimbursement model with hospital revenue. Even though this created financial alignment on group-level, alignment with the internal MSE reimbursement of physicians is lacking (ie, individual-level). The lack of individual risk-bearing due to financial misalignment at the individual level may have increased the willingness of individual physicians to engage in shared investments of the MSE. However, previous research has suggested that risk bearing on both levels should be achieved to be effective.^30^

 Fourth and lastly, an important factor contributing to hospital-MSE alignment was the shared business strategy that was present in three hospitals. Next to deepening the collaboration, the shared strategy was also remarked to be a means to justify unpopular decisions of the MSE board. This finding is in line with previous research, which suggested that involving physicians in strategic decision-making may boost alignment and may promote a cooperative environment associated with relational governance.^68,69^

###  Managerial Implications

 The current study appears to show that relational governance boosts physician unity, intensive collaboration and positive attitudes between parties. Managers may employ relational governance to draw the other party closer and developing a shared business strategy seems to promote an intensive, sustainable relationship.

 Notably, an entirely relational governance style may not be a prerequisite for an intensive collaboration. In case 4 an intensive collaboration was built with a mixed governance style, better fitting local culture. Moreover, choosing a relational stance may not be beneficial in all relationships and incongruence of governance styles may lead to increased risk of opportunistic behaviour. In case 1, where the governance style was largely contractual, adopting relational governance during a conflict led to opportunistic behaviour. Previous studies have described similar occurrences, suggesting that governance style should be congruent with parties’ perceptions and expectations.^34,45^

 Incentives were widely used, but may have been used in a suboptimal way. Two hospitals relied solely on financial penalties and only one hospital used mixed incentives, ie, financial (bonus and penalty) and non-financial (eg, supporting scientific projects). A combination of incentives may be more effective^70,71^, as solely financial incentives and penalties may not be effective long-term^72^ and may promote *gaming* behaviour.^73^ Thus, for managers aiming to use incentives as part of their governance style, it would be advisable to deploy a combination of financial and non-financial incentives.

###  Theoretical Implications

 The findings have different theoretical implications. In the proposed process model (Figure 1) we emphasized that relational characteristics may influence the governance style, which in turn may affect perceived alignment. Our findings indicate that relational characteristics (eg, previous relationship) indeed have a large impact on the governance style. However, next to relational characteristics, local context emerged as an important factor affecting the governance style and thus may be included in the model. Moreover, our process model did not consider any feedback from alignment towards governance style, whereas this has been proposed in business-to-business literature.^35^ Some cases were more comfortable relying on relational governance because they perceived a high level of alignment, indicating feedback loops indeed should be included.

 Regarding incentives, according to previous literature, efficacy of incentives used in healthcare was unclear^70,71,74-76^ and it was unknown how incentives defined on a group level (eg, MSE) influenced the behaviour of individual physicians.^74,75^ Current findings provide an interesting new perspective: although incentives were described in the contracts of different hospitals, both the hospital board and MSE board in these centres already knew that these financial penalties would never be issued in practice. Still, the hospital board deemed the use of these ‘incentives on paper’ effective, as they were a means to put a specific topic on the agenda.

 Similarly, the hospital-MSE contracts were unexpectedly found to play no important role in the subsequent relationship, even in the cases that relied more on contractual governance. In the business-to-business context the contract has been viewed as an important tool to govern the relationship regardless of the governance style.^30,37,45^ Our findings indicate that the contract is clearly not used as such in the healthcare setting. Possibly, the shared business strategy, which was present in three cases, may in part be a substitute for the contract. Future research may further investigate how contracts are used in the hospital-physician relationship.

 In previous literature there has been debate whether contractual and relational governance styles are either conflicting and competitive or whether these governance styles are complementary.^33,35,42,77,78^ In the current study, although one of the styles was dominant in most cases, use of mixed governance styles was also observed. Consequently, current findings align with previous suggestions that a governance style does not need to be fully contractual or fully relational, but rather somewhere along the continuum between contractual and relational governance.

 Focussing on agency theory, the physician has been previously described as an agent serving two principals: the hospital board and the patient.^8,41^ However, MSE board members have become the agent of yet another principal: the physicians of the MSE. A previous study suggested that the leeway of an agent may be large when multiple principals have heterogeneous interests.^79^ Our findings align with this suggestion: the board members of different hospitals accepted that the MSE board regularly made decisions in line with the interest of the MSE physicians, even though these were sometimes not in the hospitals’ interests.

###  Strengths and Limitations

 The strength of the current study is the qualitative design, being the first study to gain a deep understanding of hospital-MSE relationships in the Dutch setting. While previous studies have mostly discussed legal, organisational and fiscal aspects of the hospital-MSE relationship, we have investigated the hospital-MSE relationship from an interpersonal and collaborative perspective. Furthermore, the mixed-methods approach, using both contract analysis and interviews, has allowed us to challenge findings in either of the data sources by findings in the other. Without both sources we would not have been able to study the hospital-MSE relationship in its full complexity. In addition, the semi-structured nature of the interviews promoted discussing local context. In one case, a recent merger emerged as a ‘hot topic’ during the interview, while in another the implementation of a new electronic health record system was much discussed. Using semi-structured interviews, we gained insight into the complexity and importance of the local context.

 Some limitations of the current study ought to be discussed as well. First, the design as a qualitative multiple case study has its consequences. Whereas this study has provided a detailed insight into the hospital-MSE relationship in the five cases, these cases may not be representative for other hospitals in the Netherlands or abroad and generalizing from case study research is not straightforward.^80,81^ The sample consisted of predominantly medium to large hospitals. MSEs in smaller hospitals may have more limited resources, which might lead to a larger sense of hospital-dependency. On the contrary, an MSE with fewer physicians might create more unity and may be less challenging to manage. The *convenience *and *snowball* sampling method used may have contributed to this sample. However, this sampling approach is justifiable for the sake of gaining ‘superior access’ to sensitive information and contracts.^82^ Secondly, social desirability bias could have played a role during the interviews. If this bias is present in the current study, the hospital-MSE relationship would be more troubled than currently presented. However, measures to minimize social desirability bias were taken, such as pseudonymizing interview data.^83^ Finally, it may be considered a limitation that the analysis was performed by one researcher. However, each step of the analysis was designed jointly by both authors, and the use of multiple data sources, transparency in coding procedures and the use of a predefined contract analysis template has safeguarded the reliability of the current analysis.^84^

###  Future Research

 Different opportunities to gain further insight into the hospital-MSE relationship can be identified. Although the current study extensively studied governance in relation to perceived alignment and a possible association between relational governance and increased alignment was observed, our study has been fully exploratory and thus no causal claims can be made. Future studies may assess and test whether relational governance leads to more perceived alignment. Furthermore, whereas this study has investigated the dyadic relationship between the hospital and MSE boards, both clearly are agents in a larger network. Investigating the role of other parties such as MSE physicians, hospital-employed physicians, hospital managers, healthcare insurers and policy-makers could broaden the perspective.^35^ Moreover, it would be interesting to investigate the hospital-physician relationship and alignment in an international context.^6,7^

 The current study has investigated the hospital-MSE relationship at one point in time. However, the hospital-MSE relationship may evolve over time. Possibly, MSEs converge to one dominant organizational type. More probable, MSEs may diverge into two distinct types as in other countries: a more entrepreneurial MSE, which is exposed to business risks, and an organisational form where physicians are (quasi-)hospital-employed.^8^ Investigating the hospital-MSE relationship throughout different moments in time could provide insight into the temporal development. Hypothetically, a high level of hospital-MSE alignment and use of relational governance would make it easier to overcome disrupting events such as a merger or a pandemic.

 Lastly, hospital-MSE alignment is a means rather than an end. Eventually, alignment should lead to sustainable relationships, high quality healthcare and cost containment. Future research must point out if a greater alignment yields such ‘relational rents.’^51^

## Conclusion

 In conclusion, considerable differences in the hospital-MSE relationship were observed regarding cooperation, governance and alignment. MSE formation may have created alignment through (1) uniting physicians, (2) boosting managerial responsibility, (3) increasing financial alignment between hospital and physicians, and (4) developing a shared hospital-MSE strategy. A shared business strategy and relational governance appear to promote collaboration, alignment and possibly an intensive, long-lasting relationship. Developing a sustainable relationship between hospital and MSE is important to improve quality of care and face future challenges in healthcare.

## Acknowledgements

 We want to thank all the participants in the interviews. Without their transparency and openness during the interviews, our study could not have taken place. The authors thank Juri Matinheikki for comments made on an earlier draft of this manuscript.

## Ethical issues

 For this study, no approval by an ethical committee was required according to Dutch Law as no personal data were collected and participants were not subjected to any medical procedures. All participants consented with taping of the interviews and participated voluntarily.

## Competing interests

 Authors declare that they have no competing interests.

## Authors’ contributions

 Study conception and design: SU, EMvR; Acquisition of data: SU; Analysis and interpretation of data: SU, EMvR; Drafting of manuscript: SU; Revisions: SU, EMvR.

## Supplementary files


Supplementary file 1. Topic List Semi-Structured Interviews Hospital-MSE Alignment.

Supplementary file 2. Contract Analysis Template.

Supplementary file 3. Code Definitions.
