# Supplementary material for: Alignment in the Hospital-Physician Relationship: A Qualitative Multiple Case Study of Medical Specialist Enterprises in the Netherlands
Source: Int J Health Policy Manag. 2023 Jan 22;12:6917. doi: 10.34172/ijhpm.2022.6917 (PMC10125078; doi:10.34172/ijhpm.2022.6917)
Supplement: Supplementary file 1 — Topic List Semi-Structured Interviews Hospital-MSE Alignment. [file ijhpm-12-6917-s001.pdf]

Article title: Alignment in the Hospital-Physician Relationship: A Qualitative Multiple Case Study of Medical Specialist Enterprises in the Netherlands

Journal name: International Journal of Health Policy and Management (IJHPM)

Authors' information: Sander Ubels[1,2], Erik M. van Raaij[2,3]*

[1]Radboud University Medical Centre, Nijmegen, The Netherlands.

[2]Erasmus School of Health Policy & Management, Erasmus University, Rotterdam, The Netherlands.

[3]Rotterdam School of Management, Erasmus University, Rotterdam, The Netherlands

(Corresponding author: eraaij@rsm.nl)

Supplementary file 1. Topic List Semi-Structured Interviews Hospital-MSE Alignment

Main research question:

- How has the MSE formation contributed to alignment within the hospital?

Context before MSE – before January 2015

1. What was the organisational structure/governance regarding the physicians before January 2015?
2. Could you describe the relationship with the [*hospital/ self-employed physicians]* before 2015?
    a. What is your experience with [*hospital boards/self-employed physicians*] in previous hospitals where you have been working?
    b. What was the role of trust, opportunism and dependence?
    c. How would you characterize the previous relationship?

Organisation and activities – since MSE formation

3. What kind of MSE was formed, regarding the organisational structure?
    a. How was this organisational structure formed? Who were involved in this process?
    b. What advantages and disadvantages does the current organisational structure have?
4. Could you describe your activities with the [*hospital board/MSE*]?
    a. Does the MSE participate in strategy development?
    b. Is the MSE involved in quality and safety policy?
    c. Is the MSE involved in purchasing of medical products and equipment?
    d. Is the MSE involved with the hospital – health care insurer relationship and purchasing of care process?
    e. Does the hospital receive any incentives from the health care insurer, are these applied to the MSE?

(Shared) goals – *the following questions are about the collaboration of the hospital and MSE and their (overlapping) goals*

5. Are the goals of the hospital and MSE shared?
    a. Which goals overlap? And which do not?
6. What activities are commenced to align the goals of both parties?
    a. Has the alignment increased or decreased? What is the cause for this change?
7. Has the MSE formation contributed to alignment?
    a. In what way?
8. What role does the contract play in your collaboration with the [*hospital board/MSE*]?
    a. Does the contract stimulate alignment? Why, and how?
9. Are incentives being used to stimulate alignment?
    a. Type of incentives?
    b. Do you think incentives are effective?

Relationship – *the following part will focus on the current relationship between the hospital and the MSE boards*

10. How would you describe the current relationship with the [*hospital board/MSE*]?
    a. What is the role of trust in this relationship?
    b. Do you experience opportunism? What is its role?
    c. Could you describe the extent and role of dependency in your relationship?
    d. How would you characterise your relationship (formal/informal, at arm's length/involved, focussed on own interest/collective interest)?
11. How do you invest in the relationship with the [*hospital board/MSE*]?
12. What does the [*hospital board/MSE*] undertake to prevent opportunism?
13. Have the power relations in the hospital changed since the MSE?
14. Has the relationship between individual self-employed physicians and hospital-employed physicians changed since the MSE formation?
15. Last question: would you like to add anything regarding the hospital-MSE relationship that is important and was not discussed?
