# Supplementary material for: Alignment in the Hospital-Physician Relationship: A Qualitative Multiple Case Study of Medical Specialist Enterprises in the Netherlands
Source: Int J Health Policy Manag. 2023 Jan 22;12:6917. doi: 10.34172/ijhpm.2022.6917 (PMC10125078; doi:10.34172/ijhpm.2022.6917)
Supplement: Supplementary file 2 — Contract Analysis Template. [file ijhpm-12-6917-s002.pdf]

Article title: Alignment in the Hospital-Physician Relationship: A Qualitative Multiple Case Study of Medical Specialist Enterprises in the Netherlands

Journal name: International Journal of Health Policy and Management (IJHPM)

Authors' information: Sander Ubels[1,2], Erik M. van Raaij[2,3]*

[1]Radboud University Medical Centre, Nijmegen, The Netherlands.

[2]Erasmus School of Health Policy & Management, Erasmus University, Rotterdam, The Netherlands.

[3]Rotterdam School of Management, Erasmus University, Rotterdam, The Netherlands

(Corresponding author: eraaij@rsm.nl)

Supplementary file 2. Contract Analysis Template

Based on the items below, a contract was categorized as prevention contract or promotion contract

	**Prevention contract**	**Promotion contract**
Size (pages/attachments)	High	Low
Contract duration	Early termination	Easily extendible
Language use	Rights and obligations	Responsibilities
Perspective	Separate companies	Collective view
Incentives	Financial, penalties	Mixed, bonuses
Goals	Specific, detailed, 'to be minimally achieved'	Higher, universal level, 'to be optimally achieved'
Monitoring	High	Low
Contractual control	High	Low
Penalties	Described	Not described
Unforeseen circumstances	Procedure described	Procedure not described
Conflict resolution	Procedure described, possibly in court	Procedure not described joint resolution potential
Strategy	Little described	Joint/shared strategy
Exclusivity	Penalty when violated	Bonus when honoured

Analysis per contract based on the following items:

Item	Definition/question
**General**	
Number of pages	
Attachments: number and number of pages	
Attachments: subject	What subjects are covered in the attachments?
Parties specified	Which parties are defined in the contract?
Contract duration	Number of months/years
Continuation of contract	Does the contract describe the procedure to extend the contract?
**Financial settlement**	
Form of payment	How is payment made?<br>(Lump-sum, fixed price, ceiling)
Ex ante/ex post	Is payment negotiated ex ante or ex post?
Profit sharing	Does the contract describe procedure of sharing of profits?
Risk sharing	Does the contract describe the procedure for shared losses/risks?
Internal reimbursement	Does the contract cover the internal reimbursement model of the MSE?
**Strategy**	
Goal/mission	Does the contract describe goals/mission/vision of the parties? Are these clearly defined?
Perspective	Are the goals/mission/vision described from an individual or shared perspective?
Social norms	Are general social norms or codes of conduct described?
Hospital strategy	Does the contract describe the role of the MSE within the hospital strategy?
**Goals and incentives**	
Specific goals	Does the contract describe specific goals?
Performance indicators	Does the contract describe performance indicators? (Both quantitative as well as qualitative)
Level of detail	Are the goals described detailed/practically, or are the goals more universal?
Goal framing	Are the goals framed as minimal or optimal?
Incentives	Are incentives described linked to specific goals?
Incentive type	Financial or non-financial?
Incentive framing	Penalty or bonus?
**Contractual control**	
Audit	Are reciprocal audit procedures described?
Third party audit	Is third-party audit of the MSE described?
Performance/ benchmarking	Does the contract describe procedures to measure performance? And comparing performance with other parties/hospitals?
Periodic evaluation	Does the contract describe procedures about periodic evaluation of the agreement and assessment of performance?
Penalty	Are there penalties described?
Termination	Is the procedure of termination described? What are the grounds and term for/of termination?

Accession of individual physicians to the MSE	Does the contract describe the role of the hospital when an individual physician joins the MSE?
**Contractual coordination**	
Roles and responsibilities	Are the roles and responsibilities of both parties being described?
Tasks	Are specific tasks being described?
Tasks - Level of detail	To what detail are tasks being described?
Unforeseen circumstances	Does the contract cover the procedure for unforeseen circumstances?
Appointment of new board member	Is the reciprocal role of the hospital/MSE described when a new hospital/MSE board member is appointed?
**Conflict resolution**	
Resolution procedure	Are procedures for conflict resolution being described?
Mediation/third-party involvement	Are procedures for mediation and third-party involvement for conflict resolution stimulated?
**Health care insurer**	
Hospital agreements	Is the role of het MSE in hospital – insurer agreements being described?
Financial consequences MSE	Is the MSE involved in financial agreements between the hospital and health care insurer?
**Exclusivity**	
Exclusivity	Is there an exclusivity clause?
Confidentiality and non-competition	Is there a non-competition clause in the contract?
