# Supplementary material for: Alignment in the Hospital-Physician Relationship: A Qualitative Multiple Case Study of Medical Specialist Enterprises in the Netherlands
Source: Int J Health Policy Manag. 2023 Jan 22;12:6917. doi: 10.34172/ijhpm.2022.6917 (PMC10125078; doi:10.34172/ijhpm.2022.6917)
Supplement: Supplementary file 3 — Code Definitions. [file ijhpm-12-6917-s003.pdf]

Article title: Alignment in the Hospital-Physician Relationship: A Qualitative Multiple Case Study of Medical Specialist Enterprises in the Netherlands

Journal name: International Journal of Health Policy and Management (IJHPM)

Authors' information: Sander Ubels[1,2], Erik M. van Raaij[2,3]*

[1]Radboud University Medical Centre, Nijmegen, The Netherlands.

[2]Erasmus School of Health Policy & Management, Erasmus University, Rotterdam, The Netherlands.

[3]Rotterdam School of Management, Erasmus University, Rotterdam, The Netherlands

(Corresponding author: eraaij@rsm.nl)

Supplementary file 3. Code Definitions

Code	**Definition**
***Governance** – codes on the governance style and approach*	
Forcing	Forcing certain activities or roles
Involving	Creating involvement, importance of, way of creation involvement
Complex governance	More complex governance through MSE
Conflict resolution	The way that conflicts were resolved
Consensus	Consensus as a way of decision-making
Decisiveness	Typology of vigour/decisiveness
Dysfunctional physician	Activity: how to handle dysfunctional physician
Hierarchy	Typology/description of hierarchy and reciprocal power
Purchasing for care	Activity: how goods are being purchased
Quality and safety	Activity: quality and safety management
Decision-making	The way that decisions are being made
Source of conflict	The way that conflicts start
Strategy	Activity: developing common strategy/mission
Pace	The pace of decision-making/overriding authority
Typology conflict	When a conflict is categorized
***Context** – codes on the local context/environment*	
Culture environment	Environmental factors as explanation for collaboration/relationship
Culture hospital	Hospital culture as explanation for collaboration/relationship
Merger	Consequences of hospital mergers
Zero growth	Consequences of 'zero-growth'-policy
Context hospital	Description of hospital context, internal/external/financial/strategic
Change of board	Consequences of changes in the board
***Contract** – codes on the contract and its role in the collaboration*	
Bonus	Bonus incentives
Contract in drawer	Contract is not being used during collaboration
Detail contract	Level of detail
Advanced contract	Clear expectations of what the contract should look like
Incentives	Use of incentives
Content contract	Content of contract
Loose contract	Contract is not lived up to
Penalty	Financial penalties
Normative contract	Normative passages in contract
Development of contract	How parties developed contract
Role contract	Typology of contract and role in relationship

Obligatory contract	Contract describes duties and tasks
***Physicians** – codes on physicians and hospital-employed physicians*	
Hospital-employed	Role and identity of hospital-employed physicians
Identity physician	Views on background, motives and identity of physician
Individual specialities/physicians	The interest of individual specialties/physicians
Role staff convention	Previous role of staff convention
Role MSA	Activities of medical staff association (MSA)
***Alignment** – codes on alignment or lacking alignment*	
Shared risk	Sharing of financial risks
Financial alignment	MSE is aligned with hospital reimbursement
Alignment in quality	More alignment in quality of care
Strategic alignment	More alignment in business strategy
Alignment between physicians	More alignment between physicians
High quality of care	Having high quality of care as primary motivation
Conflicting	Conflicting interests of parties
Disunity	Disunity of parties
Reimbursement	(internal) reimbursement model
***MSE** – codes on development, role and position of MSE*	
Corporation	When the importance of organisation as corporation was highlighted
Consequences for patients	Consequence of MSE for patients
Corporation efficient	Increasing efficiency of MSE
Corporation power	Increasing power of MSE
Internal reimbursement	Internal reimbursement model of MSE
Logical corporation	When a corporation was deemed logical
Power physician	Discussing power of physicians
Mandate	Discussing authority/mandate of MSE (board)
MSE inefficient	Drawbacks of MSE
Role MSE	Role of MSE
Control	Wanting to control something
***Constitution and transition** – codes on constitution of MSE and transition to MSE*	
Goal of transition	Goal of MSE formation
Further development	Further develop collaboration/relationship
Previous organisation	Organisation before MSE formation
Previous relationship	Hospital – physician relationship before 2015
Fiscal necessity	Only fiscal necessity of MSE formation
Necessity transition	When the necessity/driving force for transition is described
Participatory model	Descriptions of participatory model
Expectations before	Internal/external expectations of hospital-MSE relationship
Conditions hospital board	If the MSE needed to meet certain conditions of the hospital board
***Relationship, perspective, approach** – normative codes on the hospital-MSE relationship*	
Informal important	Importance of informal relationship
Important decision	Description of an important decision
Captive	When captive parties are being described
Distance	Perceived distance between parties
Others' interests	Underscores individual interest of other parties
Emotion	When emotions are being described
Formal	Formal relationship
Fragile	When the relationship is described as fragile
Money important	When the importance of money is underscored
Money secondary	When money is not found important
Together	Shared perspective in conflict, decision or activity
Good collaboration	Description of good collaboration
marriage	Description of relationship/collaboration as marriage (bound)

Informal	Informal contact
Short term	Focus on short term, short-sighted
Separate companies	View of hospital and MSE as two separate companies
Power	Description of power, or consequence of power
Norms	When social norms are described
Not important	Finding something irrelavant/not important
Immature	Describing the MSE-board as immature
Pleasure	Having a nice consequence/result
Political parties	Collaboration as political parties (metaphor)
Relationship MSE- hospital board	Current hospital board – MSE relationship
Bad collaboration	Description of bad collaboration
Transparent	Increase of transparency
Responsibility	Typology of responsibility and consequence
Connected	Connected, one perspective
Cautious	Cautious behaviour
Hospital board – codes on the hospital board	
Board identity	Views on motives/position/identity of hospital board
Vulnerable	Vulnerable positioning in conflicts
Role board	Role or responsibility of hospital board
Board final responsibility	Underscoring the position of hospital board as finally responsible
***Trust** – codes on trust and distrust*	
Importance of trust	Description on importance of trust/consequences
Developing trust	Description on how trust is stimulated
Opportunism	Examples or consequences of opportunistic behaviour
Role trust	Role of trust in collaboration/relationship
Distrust	Passage on distrust and consequence
***Insurer** – codes on health care insurer and purchasing of care*	
Identity insurer	Views on the identity and role of health care insurer
Purchasing of care	Views on purchasing of care by insurer
Negotiation team	Activity: negotiating with insurer for purchasing of care
VBHC	Innovative purchasing, value-based health care
